# Impacts of opium addiction on patterns of angiographic findings in patients with acute coronary syndrome

**DOI:** 10.1038/s41598-022-19683-7

**Published:** 2022-09-08

**Authors:** Alireza Abdiardekani, Maryam Salimi, Shirin Sarejloo, Mehdi Bazrafshan, Amir Askarinejad, Amirhossein Salimi, Hanieh Bazrafshan, Shokoufeh Khanzadeh, Salar Javanshir, Armin Attar, Mohsen Esmaeili, Hamed Bazrafshan Drissi

**Affiliations:** 1 Department of Cardiologyardiology Medicine, Shiraz University of Medical Science, Shiraz, Iran; 2 Student Research Committee, Shiraz University of Medical Science, Shiraz, Iran; 3 Cardiovascular Research Center, Shiraz University of Medical Sciences, PO Box: 71348-14336, Shiraz, Iran; 4 Student Research Committee, Shahid Sadoughi University of Medical Sciences, Yazd, Iran; 5 Department of Neurology, Clinical Neurology Research Center, Shiraz University of Medical Sciences, Shiraz, Iran; 6 Student Research Committee, Tabriz University of Medical Sciences, Tabriz, Iran; 7 Islamic Azad University Medical Branch of Tehran, Tehran, Iran

**Keywords:** Cardiac device therapy, Cardiovascular biology, Interventional cardiology, Cardiology

## Abstract

Opium is one of the most abused substances in the Middle East. The effects of opium use on coronary artery disease (CAD) are a matter of debate. This study aimed to assess the association between opium use and angiographic findings as well as the complexity of CAD in patients with acute coronary syndrome (ACS) diagnosis. In this case–control study, all patients admitted for coronary angiography from 2019 to 2020 were evaluated. After applying the eligibility criteria, they were categorized into two groups opium and non-opium based on their history of opium use. Both groups were matched regarding the demographic features. The prevalence, location, and severity of obstruction of the vessels were compared between the non-opium and opium groups. The SYNTAX score was also calculated and compared between the two groups. The scores ≤ 22 are considered low risk and the higher scores are a non-low risk. P value < 0.05 is considered significant. A total of 170 patients with a mean age of 61.59 ± 9.07 years were finally enrolled in our study. Regarding the severity of vascular involvement, there was a significant difference between the non-opium and opium groups in LAD (P = 0.025), and PLV (P = 0.018) vessels. From the location points of view of obstructive coronary artery involved segments, only in the PDA (P = 0.006), and LCX (P = 0.004) vessels, a significant difference was observed. Moreover, 47.1% of opium and 30.6% of non-opium use group were in the non-low risk SYNTAX score classification which is a statistically significant difference between these two groups (P value = 0.048). Opium, as an independent risk factor for cardiovascular diseases, can have specific effects on angiographic findings in patients with acute coronary syndrome. Likewise, the complexity of CAD in opium users who undergo percutaneous coronary intervention is significantly higher.

## Introduction

Opium, after tobacco, is the most abused substance in the Middle East^1^. Opioids are commonly known for their pain relief effects, but they impact other organs like the Central nervous system (CNS), intestinal tube, lung, and heart^2,3^. Opioids can have an ischemic preconditioning effect through kappa (κ) and delta (δ) receptors in the myocytes^4^.

Numerous studies have investigated the effects of opioid abuse on the cardiovascular system, and they have offered conflicting results as some studies raise some risk factors for coronary heart disease such as Hb A1c, coagulation factor 7, fibrinogen, and CRP^5^ and therefore, declared that opium addiction would rise the mortality risk from cardiovascular events. On the other hand, several studies have reported that the prevalence of opium abuse in patients with coronary artery disease (CAD) is more than in the general population and even after considering and adjusting for other risk factors, mostly smoking, opium consumption in non-smoking groups has been recommended as an independent risk factor for CAD^4,6^.

Sadat et al.^7^ suggested that the higher levels of inflammatory factors such as interleukin 1 in opium addicts may fascinate the atherosclerosis process and therefore, opium is discussed as a predisposing factor for CAD in this study. However, other investigations have demonstrated the inverse results. They reported the opioid’s chronic consumption may lead to a reduction in the severity of CAD. The aforementioned studies have suggested that long-term opium use would reduce the intensity of CAD and the following fatal myocardial infarction^4,7–9^.

The contradictory reports regarding the role of opium use in CAD and its extension and considering the high prevalence of opium use in this region prompted us to examine the relationship between opium dependence and CAD. The study objective is the effect of opioid abuse on the severity and pattern of the coronary obstructive lesion in patients with acute coronary syndrome undergoing coronary angiography.

## Materials and methods

### Study design and participant selection

In this hospital-based case–control study, all patients for whom angiography procedure was done from March 2019 to March 2020 at Shiraz Al-Zahra Heart Hospital with the diagnosis of acute coronary syndrome were evaluated. Those who had ST-elevation myocardial infarction (STEMI) were excluded.

Before angiography, a trained examiner collected important demographic details and possible confounding variables such as sex, age, and education. Patients with risk factors such as hypertension, diabetes mellitus, history of cigarette smoking, hyperlipidemia, and obesity were excluded. Obesity was defined as body mass index (BMI) ≥ 30. According to DSM-IV criteria, all patients were assessed for opioid dependence^10^. The participants were divided into two categories, "non-users" who had no history of opium consumption (n = 85) and "active consumers" who were addicted and used opium regularly (n = 85). Both groups were matched by age and sex. Cardiovascular specialists performed all coronary angiography using standard techniques; then, an experienced cardiologist checked all angiography films. The vessels that were examined in this study included left anterior descending artery (LAD), first diagonal artery (D1), second diagonal artery (D2), left circumflex artery (LCX), posterior descending artery (PDA), right coronary artery (RCA), first obtuse marginal artery (OM1), second obtuse marginal artery (OM2), third obtuse marginal artery (OM3), left main artery (LM), and posterior left ventricular artery (PLV). In this study, patients were classified into three groups regarding the severity of vascular obstruction.

### Patient classification

Coronary artery lesions were classified into four groups normal, mild, moderate, and severe based on the amount of stenosis. Normal was attributed to the coronary artery with no lesion, mild stenosis is defined as the coronary lesion involvement of less than 50%. Between 50 and 70% of coronary involvement is considered moderate, and more than 70% of involvement is severe stenosis. Severe and moderate lesions (stenosis > 50%) are considered obstructive lesions while stenosis of less than 50% is non-obstructive.

Based on the involved number of coronary artery patients were categorized into three groups one vessel, two vessels, and three vessels. We consider the obstructive stenosis in each main vessel or its territory as the significant stenosis and classified patients into the three aforementioned groups according to the number of significantly involved coronary arteries in each patient.

Obstructive coronary artery-involved segments have been localized into three groups of proximal, middle, and distal based on the report of interventional cardiologist.

SYNTAX score was calculated online by the most recently updated version (www.syntaxscore.org). Based on the SYNTAX score the patients were divided into two groups low risk (SYNTAX score ≤ 22) and non-low risk (SYNTAX score > 22).

### Ethical consideration

These candidates were selected based on medical history, physical examination, and para-clinical results. All participants were asked to sign the written informed consent that explained all the research details and approval for the implementation of coronary angiography. The protocol was registered and approved by the Medical Ethics Committee of the Cardiovascular Research Center, Shiraz, Iran (IR.SUMS.REC.97.01.01.18389).

### Statistical analysis

Data analysis was conducted using the Statistical Package for the Social Sciences (SPSS Inc., Chicago, IL, USA), Version 26. Descriptive statistics, including mean and standard deviation, were considered. The chi-square test was used to assess the significant difference between the "opium group" and "non-opium group" based on the severity of the vessel obstruction, location of the lesion, and obstructive lesion. A p-value less than 0.05 was considered to be statistically significant.

### Ethics approval and consent to participate

All procedures performed in studies involving human participants were in accordance with the ethical standards of the institutional and national research committee and with the 1964 Helsinki declaration and its later amendments or comparable ethical standards. Approval was granted by the ethical committee of Shiraz University of Medical Sciences with the code number IR.SUMS.REC.97.01.01.18389. All the patients who agreed to participate in the trial provided written informed consent.

### Informed consent

Written Informed consent was obtained from all individual participants included in the study. The purpose of this research was entirely explained to the patients. They were assured that their information would be kept confidential by the researcher.

## Results

**Figure 1:**
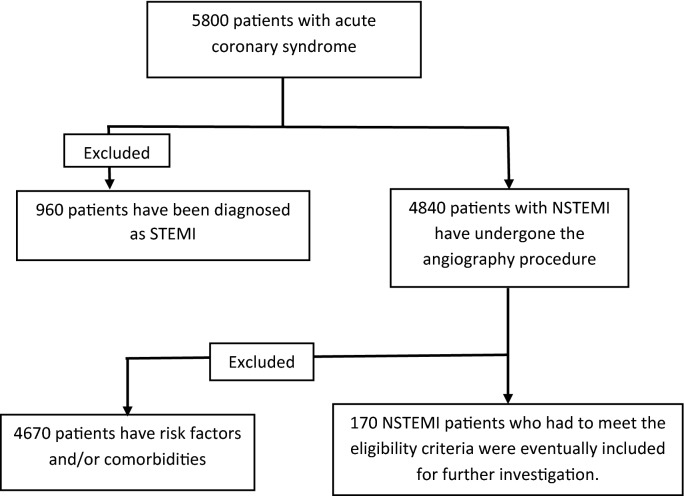
Pathway design of the study.

A total of 5800 individuals with the impression of ACS were admitted and 960 of them were diagnosed as STEMI and excluded. Among the rest, 4670 were excluded because of the aforementioned risk factors and/or comorbidities. The pathway design of the study was demonstrated in Fig. 1. A total of 170 patients with a mean age of 61.59 ± 9.07 years were finally enrolled in our study. Males constituted 120 (70.59%) while 50 (29.41%) patients were female. Notably, in the non-opium group, there were 30 females (35.29%) and 55 males (64.71%); the patients’ mean age in this group was 62.22 ± 9.31 years. In the opium group, there were 20 females (23.53%) and 65 males (76.47%), and the patients’ mean age in this group was 60.97 ± 8.61 years. Sixty-one participants (71.76%) used the Inhalation method, 19 (22.35%) used the ‘Per os’ (PO) method, and 5 (5.88%) of them used the ‘intravenous’ (IV) method. It is noteworthy that using opium was more common in men significantly (P value < 0.0001). The inhalation method was the most common opium consumption method (P value < 0.0001).

Table 1 revealed the severity of vascular involvement in the non-opium and opium groups in detail. There was no significant difference between the non-opium and opium groups in D1 (P = 0.102), D2 (P = 0.057), LCX (P = 0.100), PDA (P = 0.554), OM1 (P = 0.295), OM2 (P = 0.122), OM3 (P = 0.129), RCA (P = 0.356), and LM (P = 0.167) vessels. However, there was a significant difference between the non-opium and opium groups in LAD (P = 0.025), and PLV (0.018) vessels. The prevalence and severity of stenosis in LAD and PLV were higher in opium users.

**Table 1:** The prevalence and severity of coronary lesions in the non-opium and opium groups.

Variable	Opium group N (%) Total = 85				Non-opium group N (%) Total = 85				p-value
	Normal	mild	Moderate	Severe	Normal	Mild	Moderate	Severe	
LAD	00.0	4 (4.7)	24 (28.2)	57 (67.0)	5 (5.9)	11 (12.9)	19 (22.3)	50 (58.8)	0.025
D1	28 (32.9)	26 (30.6)	14 (16.5)	17 (20.0)	22 (25.9)	21 (24.7)	28 (32.9)	14 (16.5)	0.102
D2	68 (80.0)	8 (9.4)	5 (5.9)	4 (4.7)	61 (71.8)	6 (7.0)	3 (3.5)	15 (17.6)	0.057
LCX	18 (21.2)	28 (32.9)	24 (28.2)	15 (17.6)	19 (22.3)	16 (18.8)	24 (28.2)	26 (30.6)	0.100
PDA	45 (52.9)	13 (15.3)	11 (12.9)	16 (18.8)	49 (57.6)	7 (8.2)	11 (12.9)	18 (21.2)	0.554
RCA	15 (17.6)	20 (23.5)	19 (22.4)	31 (36.5)	16 (18.8)	14 (16.5)	14 (17.5)	41 (48.2)	0.356
OM1	36 (42.3)	24 (28.3)	8 (9.4)	17 (20.0)	25 (29.4)	25 (29.4)	12 (14.1)	23 (27.1)	0.295
OM2	60 (70.6)	14 (16.5)	5 (2.4)	6 (10.6)	72 (84.7)	6 (7.1)	3 (5.9)	3 (2.6)	0.122
OM3	79 (92.9)	2 (2.4)	00.0	4 (4.7)	82 (96.5)	3 (3.5)	00.0	00.0	0.129
LM	63 (74.1)	11 (12.9)	9 (10.6)	2 (2.4)	58 (68.2)	20 (23.5)	7 (8.2)	00.0	0.167
PLV	74 (87.1)	2 (2.3)	00.0	9 (1.6)	75 (88.2)	8 (9.4)	00.0	2 (2.4)	0.018

*LAD* left anterior descending artery, *D1* first diagonal artery, *D2* second diagonal artery, *LCX* left circumflex artery, *PDA* patent ductus arteriosus, *RCA* right coronary artery, *OM1* first obtuse marginal artery, *OM2* second obtuse marginal artery, *OM3* third obtuse marginal artery, *LM* left main artery, *PLV* posterior left ventricular artery.

Also, for the obstructive lesion, among the participants in this study, there was no significant correlation between the non-opium and opium groups in D1 (P = 0.233), D2 (P = 0.160), LCX (P = 0.097), PDA (P = 0.360), OM1(P = 0.159), OM2 (P = 0.075), OM3 (P = 0.119), RCA (P = 0.007), and LM vessels (P = 0.157); also, the results demonstrated a significant difference between two groups regarding the LAD (P = 0.009), and PLV (P = 0.002) vessels (Table 2). LAD and PLV have demonstrated the higher obstructive lesion in opium users.

**Table 2:** The prevalence of obstructive and non-obstructive lesion of the vessels in the non-opium and opium groups.

Variable	Opium group N (%) Total = 85			Non-opium group N (%) Total = 85			p-value
	Normal	Non-obstructive	Obstructive	Normal	Non-obstructive	Obstructive	
LAD	00.0	4 (4.7)	81 (95.3)	5(5.9)	11 (12.9)	69 (81.2)	0.009
D1	28 (32.9)	26 (30.6)	31 (36.5)	22 (25.9)	21 (24.7)	42 (49.4)	0.233
D2	68 (80.0)	8 (9.4)	9 (10.6)	61 (71.8)	6 (7.1)	18 (21.1)	0.160
LCX	18 (21.2)	28 (32.9)	39 (45.9)	19 (22.3)	16 (18.8)	50 (58.9)	0.097
PDA	45 (52.9)	13 (15.3)	27 (31.8)	49 (57.6)	7 (8.2)	29 (31.2)	0.360
RCA	15 (17.6)	20 (23.5)	50 (58.8)	16 (18.8)	14 (16.5)	55 (64.7)	0.514
OM1	36 (42.3)	24 (28.3)	25 (29.4)	25 (29.4)	25 (29.4)	35 (41.2)	0.159
OM2	60 (70.6)	14 (16.5)	11 (12.9)	72 (84.7)	6 (7.1)	7 (8.2)	0.075
OM3	79 (92.9)	2 (2.3)	4 (4.7)	82 (96.5)	3 (3.5)	00.0	0.119
LM	63 (74.2)	11 (12.9)	11 (12.9)	58 (68.3)	20 (23.5)	7 (8.2)	0.157
PLV	74 (87.1)	2 (2.3)	9 (10.6)	75 (88.2)	8 (9.4)	2 (2.4)	0.018

*LAD* left anterior descending artery, *D1* first diagonal artery, *D2* second diagonal artery, *LCX* left circumflex artery, *PDA* patent ductus arteriosus, *RCA* right coronary artery, *OM1* first obtuse marginal artery, *OM2* second obtuse marginal artery, *OM3* third obtuse marginal artery, *LM* left main artery, *PLV* posterior left ventricular artery.

Table 3 demonstrates the prevalence and location of the obstructive coronary artery-involved segments in the non-opium and opium groups. There was no significant difference between the non-opium and opium groups in LAD (P = 0.834), D1 (P = 0.584), RCA (P = 0.859), OM1 (P = 0.177), OM2 (P = 0.688), LM (P = 0.557) and PLV vessels (P = 0.345). On the other hand, only in the PDA (P = 0.006), and LCX (P = 0.004) vessels, a significant difference was observed. The obstructive lesions were proximal of LCX and PDA among opium users, while in the non-opium group, the lesions were on the mid part and the distal portion of the aforementioned vessels respectively.

**Table 3:** The location of obstructive coronary artery involved segments in the non-opium and opium groups.

Variable	Opium group N (%)				Non-opium group N (%)				P-value
	Total^a^	Proximal	Middle	Distal	Total^a^	Proximal	Middle	Distal	
LAD	81	49 (60.5)	27 (33.3)	5 (6.2)	69	40 (58.1)	23 (33.3)	6 (8.6)	0.834
D1	31	22 (70.9)	00.0	9 (29.1)	42	34 (80.9)	00.0	8 (19.1)	0.584
D2	9	9 (100)	00.0	00.0	18	18 (100)	00.0	00.0	–
LCX	39	28 (71.8)	4 (10.3)	7(17.9)	50	21 (42.0)	22 (44.0)	7 (14.0)	0.004
PDA	27	23 (85.2)	00.0	4 (14.8)	29	13(44.8)	2 (6.9)	14 (48.3)	0.006
RCA	50	23 (46.0)	24 (48.0)	3 (6.0)	55	22 (40.0)	26 (47.4)	2 (3.6)	0.859
OM1	25	23 (92.0)	00.0	2 (8.0)	35	30 (85.7)	00.0	5 (14.3)	0.177
OM2	11	10 (90.9)	00.0	1 (9.1)	7	6 (85.7)	00.0	1 (14.3)	0.688
OM3	4	4 (100)	00.0	00.0	0	00.0	00.0	00.0	–
LM	11	1 (9.1)	2 (18.2)	8 (72.7)	7	2 (28.6)	1 (14.3)	4 (57.1)	0.557
PLV	9	8 (88.8)	00.0	1 (11.2)	2	1 (50.0)	00.0	1 (50.0)	0.345

*LAD* left anterior descending artery, *D1* first diagonal artery, *D2* second diagonal artery, *LCX* left circumflex artery, *PDA* patent ductus arteriosus, *RCA* right coronary artery, *OM1* first obtuse marginal artery, *OM2* second obtuse marginal artery, *OM3* third obtuse marginal artery, *LM* left main artery, *PLV* posterior left ventricular artery.

^a^The total number of involvements has been mentioned for each vessel.

Among the opium group, all patients had at least one obstructive lesion; the number of one, two, and three involved vessels were 3 (3.5%), 37 (43.5%), and 45 (52.9%) cases, respectively. In the non-opium use cases, 13 (15.2%) had no obstructive lesion and the number of one, two, and three involved vessels were 6 (7.1%), 26 (30.6%), and 40 (47.1%), respectively. Our findings revealed that three-vessel disease was more frequent in both opium (45; 52.94%) and non-opium (40; 47.05%) groups which were not statistically significant among the two groups.

The SYNTAX score was calculated for obstructive lesions. Based on our results, 45 (52.9%) cases in the opium user group had a SYNTAX score ≤ 22, and 42 (47.1%) of them had a score of 23 or more. The aforementioned amount for those non-opium cases who had an obstructive lesion (72 cases) was 50 (69.4%) and 22 (30.6%) respectively.

In other words, 47.1% of opium use and 30.6% of non-opium use group were in the non-low risk SYNTAX score classification which is a statistically significant difference between these two groups (P value = 0.048).

## Discussion

CAD is the primary cause of death in developed and developing countries^11,12^. The impact of opium addiction on CAD is a matter of debate in the literature.

The opioid receptor system includes kappa (κ), mu (μ), and delta (δ). Their endogenous opioid ligands, such as endorphin, dynorphins, and enkephalins, can have various effects on the heart, such as ischemic preconditioning, disruption of myocardial perfusion, and increased infarct size^13^. Schultz et al. in animal models have shown that there are many receptors on the heart for opium which could have protective or negative effects on the heart^14^. Therefore, the effect of opioids depends on the activation of the receptor subtype^15,16^. It has been stated that chronic opioid intake has a positive influence on the heart because of releasing endogenous substances such as P, adenosine, calcitonin-generated peptide, and adenylyl cyclase^16^. On the other hand, stimulation of Kappa opioid receptors can lead to disruption of myocardial perfusion and increase the infarct size^17^. It seems that kappa and delta receptors have a more prominent role^18^.

Stimulation of inflammatory factors and disturbance in coagulation are two main effects of opium that lead to atherosclerosis. Higher C-reactive protein and fibrinogen levels detected in opium addicts can cause a higher rate of CAD in these people^19^.

This supports previous findings in the study of Kazerani et al. in 2014 that indicated LAD was the most common artery involved in angiographic findings^11^. In 2008–2009, Abdollahi et al. showed that the highest percentage of coronary artery lesions was observed in the LAD, RCA, and LCX vessels^20^.

The association between opium and cardiovascular disease is interesting to mention. The opium users had a more prevalent vascular involvement as well as the obstructive lesion in LAD and PLV. The location of obstructive lesions was significantly different between the two groups in LCX and PDA vessels. Amon the opium groups, the lesions were commonly found in the proximal part of the aforementioned vessels among opium users, while they have been found in the middle and distal part of LCX and PDA, respectively. The prevalence of the non-low risk SYNTAX score (> 22) was higher in opium users. The most remarkable result from the data is that opium, as an independent risk factor for coronary artery disease, can have specific vascular involvement patterns.

Masoomi et al.^21^ have claimed that opium abuse is a predictive independent risk factor for ischemic stroke and deep vein thrombosis development. Moreover, Sadeghian et al.^22^ completed a study among the 2405 admitted patients in the angiography ward and established the 13.4% prevalence of opium consumption. Their findings displayed a direct relation between angiography-defined CAD and opium consumption. The strong correlation between the severity of CAD and opium use was also proved in this study. In another study a significant correlation between opium consumption in men and the prevalence of CAD presence among 940 younger patients^23^.

Asgary et al.^19^ displayed the atherogenic negative effect of opium addiction. It is important to note that a relation between CAD and opium addiction did not verify a direct correlation if confounding factors were not considered (e.g., socioeconomic status, education, etc.); it may be attributed to the poorer condition of opium users and that they were also at other risks of CAD. Nutritional problem is another confounding factor because of the increasing effect of opium on appetite and weight gain^24^. Sadeghian et al. analyzed 4398 patients who had managed isolated CABG and revealed a 15.6% of opium dependency. Therefore, he displayed the negative opium dependency effects on the outcomes of CABG^25^.

On the other hand, Najafipour et al.^26^ and Davoodi et al.^27^ believed that opium addiction would not increase the presence of CAD or deteriorate it. They have claimed that the higher mortality rate in these groups is attributed to the fact that some addict patients use opium to get pain relief. This will relieve the chest pain and cause somnolence which is followed by an increase in the elapsed time from the onset of symptoms to hospital admission.

Based on the SYNTAX score, we found that the complexity of CAD in opium users who undergo percutaneous coronary intervention is significantly higher and their prognosis would be lower. Contrary to the increasing effect of opium that has been found in our study, Moezi et al.^28^ have reported a significantly lower SYNTAX score among opium users.

From all stated above, it seems that the consumption of opioids could be an independent risk factor for CAD. It affects both anterior and posterior heart-related vessels. It is highly recommended that public health officials and politicians should prepare several projects to increase consciousness and eventually change the general population's mentality. Public health authorities should also warn people against opioid use and its disadvantages. Health practitioners should be aware of the potential impacts of using opioids on public health.

### Limitation

Our research had some limitations. The best epidemiological analysis to accentuate the objectives of the present research was the cohort, and our model was a case–control one. More studies and research are required to establish the impact of various forms of opium and their specific doses on cardiovascular diseases.

### Conclusion

This study concluded that opium was an independent risk factor with specific effects on angiographic findings in cardiovascular patients. Both LAD and PDA are significantly more affected by an obstructive lesion in opium users. In LCX and PDA the obstructive lesions were significantly desired to be in the proximal part in opium users, contrary to the middle and distal desire in opium users regarding the aforementioned vessels respectively. Furthermore, the complexity of CAD in opium users who undergoing percutaneous coronary intervention is significantly higher.

## Data Availability

The data that support the findings of this study are available from Hamed Bazrafshan (corresponding author) but restrictions apply to the availability of these data, which were used under license for the current study, and so are not publicly available.

## Abbreviations

CNS: Central nervous system
CAD: Coronary artery disease
LAD: Left anterior descending artery
D1: First diagonal artery
D2: Second diagonal artery
LCX: Left circumflex artery
PDA: Posterior descending artery
RCA: Right coronary artery
OM1: First obtuse marginal artery
OM2: Second obtuse marginal artery
OM3: Third obtuse marginal artery
LM: Left main artery
PLV: Posterior left ventricular artery
PO: Per os
IV: Intravenous
